# Mechanosensitive piezo1 calcium channel activates connexin 43 hemichannels through PI3K signaling pathway in bone

**DOI:** 10.1186/s13578-022-00929-w

**Published:** 2022-12-01

**Authors:** Yan Zeng, Manuel A. Riquelme, Rui Hua, Jingruo Zhang, Francisca M. Acosta, Sumin Gu, Jean X. Jiang

**Affiliations:** 1 Department of Biochemistry and Structural Biology, University of Texas Health Science Center, San Antonio, TX USA; 2 The Second Xiangya Hospital, Central South University, Changsha, Hunan China

**Keywords:** Mechanical loading, Piezo1, Cx43 hemichannels, Ca^2+^ signal, PI3K-Akt pathway

## Abstract

**Background:**

Mechanical loading promotes bone formation and osteocytes are a major mechanosensory cell in the bone. Both Piezo1 channels and connexin 43 hemichannels (Cx43 HCs) in osteocytes are important players in mechanotransduction and anabolic function by mechanical loading. However, the mechanism underlying mechanotransduction involving Piezo1 channels and Cx43 HCs in osteocytes and bone remains unknown.

**Results:**

We showed that, like mechanical loading, Piezo1 specific agonist Yoda1 was able to increase intracellular Ca^2+^ signaling and activate Cx43 HCs, while Yoda1 antagonist Dooku1 inhibited Ca^2+^ and Cx43 HC activation induced by both mechanical loading and Yoda1. Moreover, the intracellular Ca^2+^ signal activated by Yoda1 was reduced by the inhibition of Cx43 HCs and pannexin1 (Panx1) channels, as well as ATP-P2X receptor signaling. Piezo1 and Cx43 HCs were co-localized on the osteocyte cell surface, and Yoda1-activated PI3K-Akt signaling regulated the opening of Cx43 HCs. Furthermore, Cx43 HCs opening by mechanical loading on tibias was ablated by inhibition of Piezo1 activation in vivo.

**Conclusion:**

We demonstrated that upon mechanical stress, increased intracellular Ca^2+^ activated by Piezo1 regulates the opening of HCs through PI3K-Akt and opened Cx43 HCs, along with Panx1 channels, and ATP-P2X signaling sustain the intracellular Ca^2+^ signal, leading to bone anabolic function.

**Supplementary Information:**

The online version contains supplementary material available at 10.1186/s13578-022-00929-w.

## Background

Mechanical signals play a critical role in bone homeostasis and remodeling, a lifelong process [1]. Osteocytes, the most abundant bone cell type embedded in the bone matrix, have long dendritic processes that connect adjacent osteocytes and communicate with osteoblasts and osteoclasts, making them the predominant cell type in sensing and transducing mechano-stimuli [2, 3]. When subjected to fluid flow shear stress (FFSS) in vitro, one of the most pivotal events that osteocytes undergo is the secretion of several signaling molecules, such as IP3 and Ca^2+^ from intracellular stores, together with the extracellular release of intracellular metabolites such as prostaglandin E_2_ (PGE_2_), ATP, and nitric oxide (NO) [3]. Ca^2+^ release from intracellular stores is the earliest event and is crucial for bone homeostasis [4]. Connexin channels, including gap junctions and hemichannels (HCs), which allow the passage of ions and molecules (< 1.2 kDa), serve as a common portal for the intracellular and extracellular exchange, respectively, of small metabolites such as second messengers (ATP, PGE_2_, NAD^+^, glutamate) and ions (Ca^2+^, Na^+^, and K^+^) [5]. Connexin 43 (Cx43) is the most ubiquitously expressed Cx, and gap junctions and HCs formed by Cx43 facilitate networking among different cell types, especially osteocytes [6, 7]. Osteocyte signals induced by mechanical stimulation include the release of ATP and PGE_2_ through Cx43 HCs [8, 9], and an increase of intracellular Ca^2+^ is shown to induce Cx43 HC opening and thus the release of these molecules [10, 11]. Extracellular PGE_2_ is a major mediator that promotes new bone formation in response to mechanical loading [12, 13], which highlights the importance of Cx43 channels in bone remodeling in response to mechanical signals [14, 15]. Moreover, PI3K-Akt has been reported as a major signaling pathway in regulating the opening of Cx43 HCs [16, 17].

During the last decade, Piezo proteins were identified as mechanosensitive ion channel subunits, and the mechanosensitive function and mechanisms of Piezo ion channels, Piezo1/2, have been unveiled [18, 19]. Piezo1 and Piezo2 are expressed in a wide range of mechanically sensitive cells. Piezo1 is primarily expressed in nonsensory tissues exposed to fluid pressure and flow, whereas Piezo2 is predominantly found in sensory tissues that respond to touch [19]. Mechanical force alters the lateral tension in the membrane bilayer or creates a hydrophobic mismatch that can open the pore [20]. Piezo channels also display locally distinct membrane curvature, thickness, and tension, thereby exaggerating their gating efficiency [21]. Moreover, a functional study in cells revealed Yoda1 as an agonist for Piezo1, making it a key compound tool in studying Piezo1 function and regulation [22, 23]. Recent studies have shown that Piezo1 is abundantly expressed in osteocytes [24]. In addition, Piezo1 functions as a dominant mechanotransducer for conducting mechanical stimuli to osteoblasts and determines mechanical-load-dependent bone formation [24, 25].

In the present study, we unveiled the functional and structural relationship between mechanosensitive Piezo1 Ca^2+^ channels and Cx43 HCs in osteocytes. Both in vivo and *in vitr*o studies showed that the opening of Cx43 HCs is regulated by Piezo1 activation upon mechanical loading or Yoda1. In addition, intracellular Ca^2+^ increase by mechano-activated Piezo1 is involved in the regulation of Cx43 HCs and pannexin 1 channels, and HC opening is regulated by activated PI3K signaling. Moreover, Piezo1 and Cx43 HCs are co-localized on the cell surface of osteocytes, and FFSS enhances the co-localization.

## Materials and methods

### Cell culture and generation of Rosa26 and Cx43 KD MLO-Y4 cell line

Osteocytic MLO-Y4 cells derived from murine long bones were provided by Dr. Lynda Bonewald at Indiana University School of Medicine. Lentiviral-mediated CRISPR/Cas9 genome editing technology using lentiCRISPRv2:Cx43-sgRNA or ROSA26-sgRNA was applied to generate Cx43 KD and Rosa26 control MLO-Y4 cells as previously described [26]. These cells were cultured on collagen-coated (rat tail collagen type I, 0.15 mg/ml) surfaces and were grown in medium (α-MEM, HCO_3_^−^) supplemented with 2.5% fetal bovine serum and 2.5% bovine calf serum and incubated in a 5% CO_2_ incubator at 37 °C as described previously [27].

### Fluid flow shear stress and in vitro dye uptake assay

FFSS experiments were conducted as previously described [28]. Briefly, FFSS was created by parallel plate flow chambers separated by a gasket of defined thickness with gravity-driven fluid flow using a peristaltic pump. Shear stress levels of 2, 4, 8, and 16 dynes/cm^2^ were generated, and a cell surface area of 5 cm^2^ was used for dye uptake assay. MLO-Y4 cells were pre-incubated with Cx43(E2) antibody, generated and affinity-purified as previously described [29], ^10^Panx (#33-481, Tocris Bioscience, Bristol, UK), Dooku1 (#6568, Tocris Bioscience, Bristol, UK), BAPTA-AM (#B1205, Invitrogen, Carlsbad, CA, USA), apyrase (#A6237-100UN, Sigma-Aldrich, St. Louis, MO, USA), and LY294002 (#NC9642062, Cayman Chemical, Ann Arbor, MI, USA). The cells were then subjected to FFSS (2, 4, 8, and 16 dynes/cm^2^, 10 min), followed by dye uptake assay. In dye uptake assay, cells were incubated with 10 μM ethidium bromide (EtBr) (Mw: 394 Da) and FITC-Dextran (Mw: 10 kDa) at room temperature (RT) for 5 min and FITC-Dextran was used to detect the apoptotic and unhealthy cells. The FITC-Dextran-positive cells were excluded for quantification of dye uptake level. The treatment with Yoda1 (#5586, Tocris Bioscience, Bristol, UK) was performed in the presence of the dyes. Cells were rinsed with PBS and fixed with 2% paraformaldehyde, and the mean intensity of the nuclear region was determined using NIH ImageJ software (NIH, Bethesda, MD, USA) [8].

### Live cell calcium imaging

MLO-Y4 cells were cultured at a total of 2.5 × 10^5^ cells on a collagen-coated 35 mm culture dish with an inner 25-mm diameter circle glass slide 48 h prior to live Ca^2+^ imaging. Before imaging capturing, cells were incubated with Cx43(E2) antibody, ^10^Panx, Dooku1, BAPTA-AM, oATP (#71997-40-5, Sigma), and apyrase (#9000–95-7, Sigma) at RT for 30 min, followed with Fluo-4-AM (#F14201, Invitrogen), a Ca^2+^ indicator, at 37 ℃ for 40 min. The culture medium was then rinsed and replaced with recording medium (α-MEM, 10 mM HEPES, PH 7.4), and the glass slide was placed in the Coverslip Cell Chamber (#SC15012, AirekaCells, HK, China). The chamber was placed on the objective stage of the microscope and set still before capturing images. Ca^2+^ signaling (488 nm) of the MLO-Y4 cells was imaged using a Nikon sweptfield laser scanning confocal microscope (Nikon, Tokyo, Japan) with a 40X objective. The entire imaging process lasted 600 s, and each image was taken with a 2-s interval, and Yoda1 was loaded at 240 s. For sequential inhibition of Cx43 HCs or Panx1 Channel, Cx43(E2) antibody or ^10^Panx was added at 360 s. The BAPTA-AM was added at 360 s. For the recording of Ca^2+^ signal in mechanically loaded MLO-Y4 cells, the cells cultured on coverslips were then placed in the FFSS chamber and connected with the fluid circulation system, and live calcium images were captured at 900 s with FFSS (8 dynes/cm^2^) stimulation applied at 300 s and lasted for 300 s. The entire process lasted for 900 s with 3 phases (300 s each) rest, stimulation, and recovery.

### Cell surface staining

MLO-Y4 cells cultured on collagen-coated glass slides were subjected to FFSS (16 dynes/cm^2^) for 1 h or Yoda1 for 30 min and rinsed 3 times with cold (4 ℃) DPBS. The live cells on the slides were incubated with rabbit polyclonal anti-Cx43(E2) antibody (1:100 dilution) in recording medium supplemented with 2.5% fetal bovine serum and 2.5% bovine calf serum at 4 ℃ for 90 min. After rinsing 3 times with cold DPBS supplemented with 1 mM Ca^2+^, cells were fixed with 2% paraformaldehyde for 10 min, followed by blocking with donkey blocking buffer (2% donkey serum, 2% fish skin gelatin, 0.025% Triton X-100 and 1% bovine serum albumin in PBS). The cells were then incubated with 1:100 dilution mouse monoclonal anti-Piezo1 antibody (clone 2–10 ThermoFisher Scientific, Waltham, MA, USA) at 4 ℃, overnight. The labeling by primary antibodies was performed by sequential incubation of secondary antibodies first with donkey anti-mouse Alexa Fluor 488-conjugated (#NC0241229, Jackson ImmunoResearch, West Grove, PA, USA) for 1 h and then with donkey anti-rabbit Alexa 594 (#NC0303478, Jackson ImmunoResearch, West Grove, PA, USA) for 1 h. Slides were mounted and sealed for microscopic analysis. Confocal sectional images were taken with an optical microscope (BZ- X710, KEYENCE, Itasca, IL, USA) using the appropriate filters.

### Preparation of cell lysates and Western blot

LY-294002/Yoda1 treated MLO-Y4, Cx43 KDa, and Rosa26 MLO-Y4 cells were collected and lysed with lysis buffer (5 mM Tris, 5 mM EDTA/EGTA, 0.6% sodium dodecyl sulfate (SDS) and proteinase inhibitors) and then ruptured by pipetting through a 20-gauge needle. Cell lysates with Cx43 KD or Rosa26 control were ultracentrifuged at 45,000 g for 45 min at 4 ℃ to prepare crude membrane extracts. Protein concentration of protein lysates was determined by Micro BCA Protein Kit (Thermo Scientific, Rockford, IL, USA). 30 µg proteins were boiled in SDS sample loading buffer (250 mM Tris–HCl, pH 6.8, 10% SDS, 30% (v/v) glycerol, 10 mM DTT, 0.05% (w/v) bromophenol blue), subjected to 10% SDS–polyacrylamide gel electrophoresis, and electroblotted onto a nitrocellulose membrane. Membranes were blocked with donkey serum blocking buffer at RT for 1 h, then incubated with a 1:1000 dilution of rabbit polyclonal anti-Akt antibody (#9272, Cell Signaling Technology, Danvers, MA, USA) and rabbit polyclonal anti- phosphorylated-AKT Ser473 antibody (#9271, Cell Signaling Technology) (1:300 dilution) for LY-294002/Yoda1 treated MLO-Y4 cell samples or incubated with 1:500 dilution of rabbit polyclonal anti-Cx43(CT) antibody or 1:150 of mouse monoclonal anti-Piezo1 antibody (#MA5-32876, Invitrogen) for Cx43 KD and Rosa26 samples. Primary antibodies were detected with goat anti-rabbit IgG conjugated IRDye® 800CW and goat anti-mouse IgG conjugated IRDye® 680RD (1:15 000 dilutions) using a Licor Odyssey Infrared Imager (Lincoln, NE, USA). The band intensity was quantified by densitometry using NIH ImageJ software.

### Immunoprecipitation

MLO-Y4 cell lysates were resuspended in immunoprecipitation (IP) buffer (0.1 M NaCl, 15 mM EDTA, 15 mM EGTA 0.02% Na azide, 0.02 M Na borate, 10 mM NEM, 2 mM PMFS, pH 8.5). Lysates were precleared with protein A/G plus-agarose beads (NC9371547, Santa Cruz, CA, USA) and pelleted by centrifugation at 7000 rpm at 4 ℃ for 5 min. Supernatants (precleared lysates) were then incubated with rabbit polyclonal anti-Piezo1 antibody (15,939–1-AP, Proteintech, Rosemont, IL, USA) overnight at 4 ℃, followed by precipitation of the complexes after incubation with protein A/G plus-agarose beads at 4 ℃. Immunoprecipitants were separated on 10% SDS–polyacrylamide gel and immunoblotted with mouse monoclonal anti-Cx43 (SAB4200730, Sigma) (1:200 dilution) or mouse monoclonal anti-Piezo1 antibody (MA5-32,876, Invitrogen) (1:500 dilution).

### Tibia compression and in vivo dye uptake assay

We subjected 15-week-old male C57Bl/6 mice to mechanical loading through tibial compression using a loading setup established in our laboratory [16, 30, 31]. Mice were subjected to tibial loading at a frequency of 2 Hz using a haversine waveform for 600 cycles with a constant force of 8.86 N at the left tibia. 15-week-old male C57Bl/6 mice were injected intraperitoneally with Cx43(M1) antibody, control mouse IgG, 5 mg/kg Dooku1, or vehicle DMSO as control 4 h prior to intravenous tail injection of 200 mg/kg Evans Blue. Mice were subjected to intraperitoneal injection of 2.5 mg/kg Yoda1, or cyclic tibia compression 30 min after the injection of the dye, and perfusion with 4% paraformaldehyde and tissue collected 2 h or 1 h post Yoda1 administration or mechanical loading. Mice were housed in a temperature-controlled room with a 12-h light/12-h dark cycle and under specific pathogen-free conditions in the Institutional Lab Animal Research facility at the University of Texas Health Science Center at San Antonio (UTHSCSA). All described animal protocols were reviewed and approved by UTHSCSA Institutional Animal Care and Use Committee (IACUC), in accordance with policies dictated by the Office of Animal Welfare at the NIH, USA.

### Statistical analysis

GraphPad Prism8 statistics software (GraphPad, San Diego, CA, USA) was used for statistical analysis. Student-Newman Keul’s test and One-way ANOVA were used for two or multiple comparison analyses. Two-way ANOVA with post hoc Tukey test was used to assess the difference between multiple groups with two variables. Data were shown as mean ± SEM, and asterisks indicate the degree of significant differences compared with the controls, *p < 0.05, **p < 0.01, ***p < 0.001, ****p < 0.0001.

## Results

### Dose and time-dependent activation of Cx43 HCs by Piezo1 agonist Yoda1

**Fig. 1:**
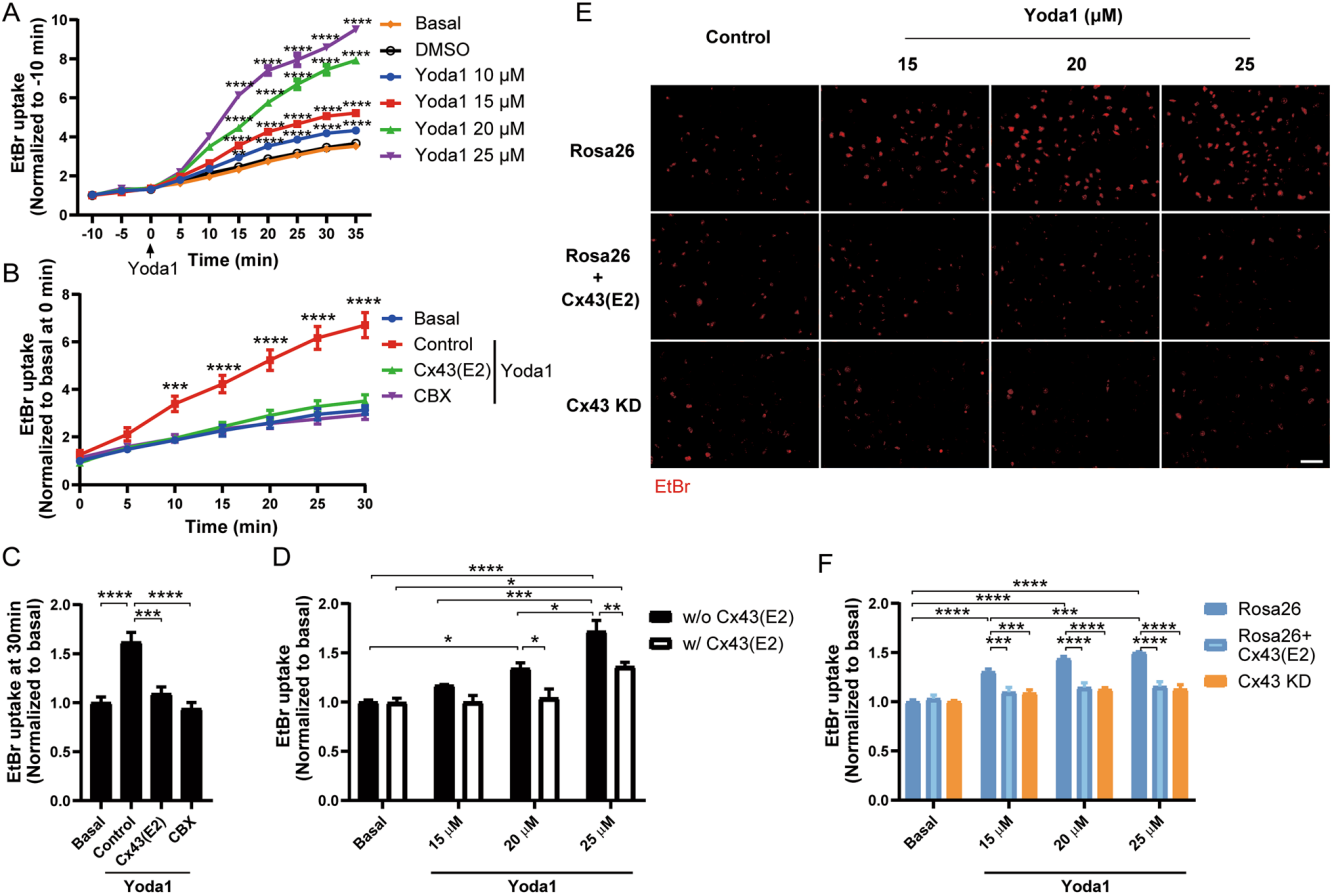
Piezo1 agonist Yoda1 induces the opening of Cx43 HCs in a dose and time-dependent manner. **A** Time-lapse EtBr dye uptake level in MLO-Y4 cells (5 min interval) with different concentrations of Yoda1 (10, 15, 20, and 25 μM) treatment. **B** Time-lapse EtBr dye uptake level in MLO-Y4 cells (5 min interval) pre-incubated with 2 μg/mL Cx43(E2) antibody or 100 μM carbenoxolone (CBX), followed by 20 μM Yoda1 treatment. **C** EtBr dye uptake level at 30 min after Yoda1 application in (**B**). **D** EtBr dye uptake level in MLO-Y4 cells pre-incubated with 2 μg/mL Cx43(E2) antibody, followed by different concentrations of Yoda1 (15, 20, and 25 μM) stimulation. **E** Representative images showing EtBr dye uptake in Rosa26 and Cx43 KD MLO-Y4 cells pre-incubated with 2 μg/mL Cx43(E2) antibody, followed by treatment with varying concentrations of Yoda1 (15, 20, and 25 μM), scale bar = 20 μm. **F** Quantification of EtBr dye uptake level (**E**). Data are shown as mean ± SEM. n = 3, *p < 0.05, **p < 0.01, ***p < 0.001, ****p < 0.0001. Statistical analysis was performed using One-way ANOVA for multiple comparison analysis in panel **C** and Two-way ANOVA with post hoc Tukey to assess the difference between multiple groups in panels **A**, **B**, **D**, and **F**

Both Cx43 HCs and Piezo1 channels expressed in osteocytes are mechanosensitive channels; however, the functional relationship of these two channels has not been assessed. MLO-Y4 cells were treated with different concentrations (10, 15, 20, and 25 μM) of Yoda1, and time-lapse images with 5 min intervals of EtBr fluorescence were taken. The cell uptake of EtBr indicated an increase in the membrane permeability. The apoptotic, unhealthy cells were assessed with FITC-Dextran, a dye with Mw of 10 kDa which is impermeable to HCs (< 1.2 kDa) but can be taken up by apoptotic, dying cells. We observed that FITC-Dextran-positive cells were overall very low as shown in Additional file 1: Fig. S1. Compared with basal and vehicle groups, the EtBr uptake level increased over time in a dose-dependent manner in all Yoda1-treated groups (Fig. 1A). To investigate whether the uptake of EtBr was specifically through Cx43 HCs, we used Cx43(E2) antibody, an inhibitor that specifically blocks Cx43 HCs [29, 32]. Time-lapse experiment showed that pre-treatment with Cx43(E2) antibody followed by 20 μM Yoda1 treatment significantly reduced EtBr uptake in MLO-Y4 cells. The reduction of dye uptake was also observed in cells pretreated with 100 μM carbenoxolone (CBX), an unspecific inhibitor for Cx and Panx channels (Fig. 1B, C) [33]. To further define the relationship of Cx43 HCs and Yoda1 in regard to dye uptake, we pretreated MLO-Y4 cells with HC-blocking Cx43(E2) antibody followed by different concentrations (15, 25, and 25 μM) of Yoda1 treatment. We found that blocking Cx43 HCs was able to reduce the dye uptake in all Yoda1 treated groups (Fig. 1D). To further confirm the role of Cx43, we knocked down Cx43 in MLO-Y4 cells using CRISPR/Cas9, and Rosa26 was used as a control (Additional file 1: Fig. S2). Knocking down Cx43 (Cx43 KD) significantly inhibited EtBr uptake compared with Rosa26-expressing control cells treated with different concentrations (15, 20, and 25 μM) of Yoda1, and the extent of the inhibition was similar to that by Cx43(E2) antibody. Moreover, either knockdown of Cx43 or inhibiting HC by Cx43(E2) antibody reduces dye uptake to basal level at higher concentrations of Yoda1 (Fig. 1E, F). This result suggested that the increase of membrane permeability observed by Yoda1-induced Piezo1 activation contributed mainly to the opening of Cx43 HCs in MLO-Y4 cells.

### Piezo1 antagonist, Dooku1 inhibits mechanical loading induced Piezo1 activation and Cx43 HCs opening

**Fig. 2:**
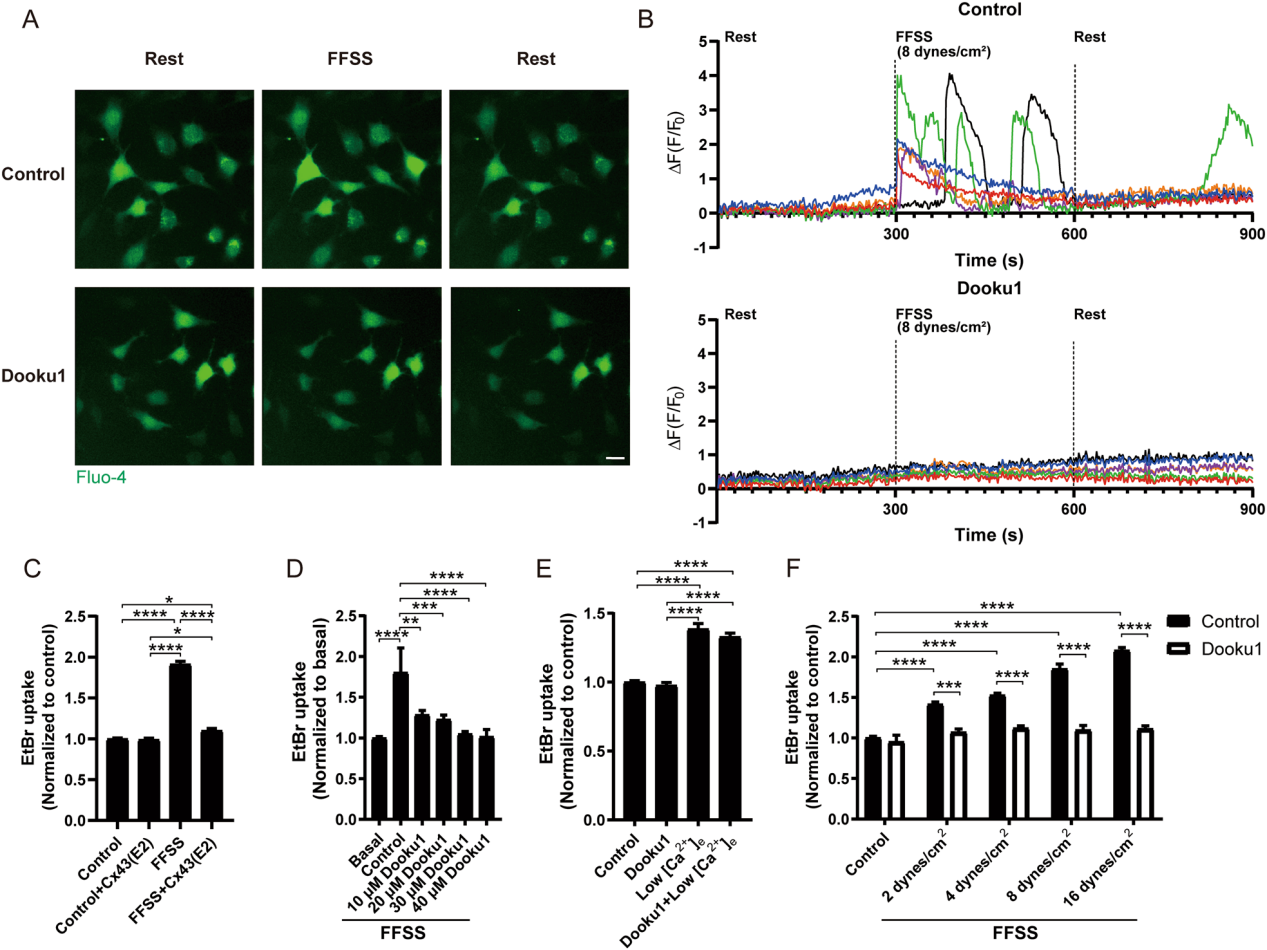
Dooku1 inhibits FFSS induced Piezo1 activation and Cx43 HCs opening. **A** MLO-Y4 cells were pre-incubated with 40 μM Dooku1 for 30 min, then placed in the FFSS chamber, fixed on the objective table of Nikon Swept Field confocal microscope, and live images were taken at 2 s intervals for a total 15 min. Representative calcium images, first images of each phase were presented, scale bar = 20 μm; **B** Real-time quantification of calcium signals in Dooku1 pre-incubated and control groups, each curve represented one cell, n = 6 per group, **A** and **B** are representative images and data of three independent experiments. **C** EtBr dye uptake level in MLO-Y4 cells pre-incubated with 40 μM Dooku1, followed by low Ca^2+^ medium treatment; **D** EtBr dye uptake level in MLO-Y4 cells pre-incubated with different concentrations of Dooku1 (10, 20, 30, and 40 μM), followed by FFSS (16 dynes/cm^2^, 10 min) stimulation; **E** EtBr dye uptake level in MLO-Y4 cells pre-incubated with 40 μM Dooku1, followed by different magnitudes of FFSS (2, 4, 8, and 16 dynes/cm^2^, 10 min) treatment. **F** EtBr dye uptake level in MLO-Y4 cells pre-incubated with 2 μg/mL Cx43(E2) antibody, followed by FFSS (16 dynes/cm^2^, 10 min) stimulation. Data are shown as mean ± SEM, **C**–**F** n = 3. *p < 0.05, **p < 0.01, ***p < 0.001, ****p < 0.0001. Statistical analysis was performed using One-way ANOVA for multiple comparison analysis for panels **C**, **D**, and **E** and Two-way ANOVA for panel F

A Piezo1 antagonist, Dooku1, was used to assess the effect of Piezo1 activation on Cx43 HCs in osteocytes. MLO-Y4 cells pre-incubated with Dooku1 and Fluo-4-AM, a free Ca^2+^ indicator, were subjected to 5-min FFSS (8 dynes/cm^2^), and live-cell images were captured. The representative images, which are the first images of each phase in the pretreated Dooku1 and control groups, showed dramatically increased levels of intracellular Ca^2+^ after FFSS stimulation. The intracellular Ca^2+^ rise was blocked significantly with Dooku1 in the recording media (Fig. 2A). Real-time quantification of Ca^2+^ signals confirmed that compared to non-treated control, Dooku1 inhibited FFSS-induced Ca^2+^ signals dramatically, suggesting the activation of Piezo1 by mechanical loading (Fig. 2B). Cx43(E2) antibody inhibited FFSS-induced EtBr uptake, confirming that FFSS-induced dye uptake was primarily mediated through Cx43 HCs (Fig. 2C). Dooku1, similar to Cx43(E2) antibody, inhibited FFSS-induced dye uptake in a dose-dependent manner in MLO-Y4 cells following FFSS (16 dynes/cm^2^, 10 min) (Fig. 2D). To confirm that Dooku1 specifically inhibited Piezo1 channels and did not directly affect HCs in the absence of mechanical loading, we evaluated the effect of Dooku1 over Cx43 HCs opening induced by Ca^2+^-depleted recording medium in MLO-Y4 cells. This condition induces the opening of functional HCs present at the cell surface [34]. We observed that, unlike mechanical stimulation, Dooku1 could not inhibit low extracellular Ca^2+^ (low [Ca^2+^]e)-induced dye uptake (Fig. 2E). The level of membrane permeabilization (dye uptake) was correlated with the magnitude of FFSS and 40 μM Dooku1 was able to inhibit dye uptake in all groups (Fig. 2F). These results showed that inhibition of mechanical stimuli induced Piezo1 activation by Dooku1 impedes the opening of Cx43 HCs in osteocytes, suggesting the importance of Piezo1 channels in the activation of Cx43 HCs.

### Mechanical loading activated Piezo1 and Ca^2+^ influx induce the opening of Cx43 HCs

**Fig. 3:**
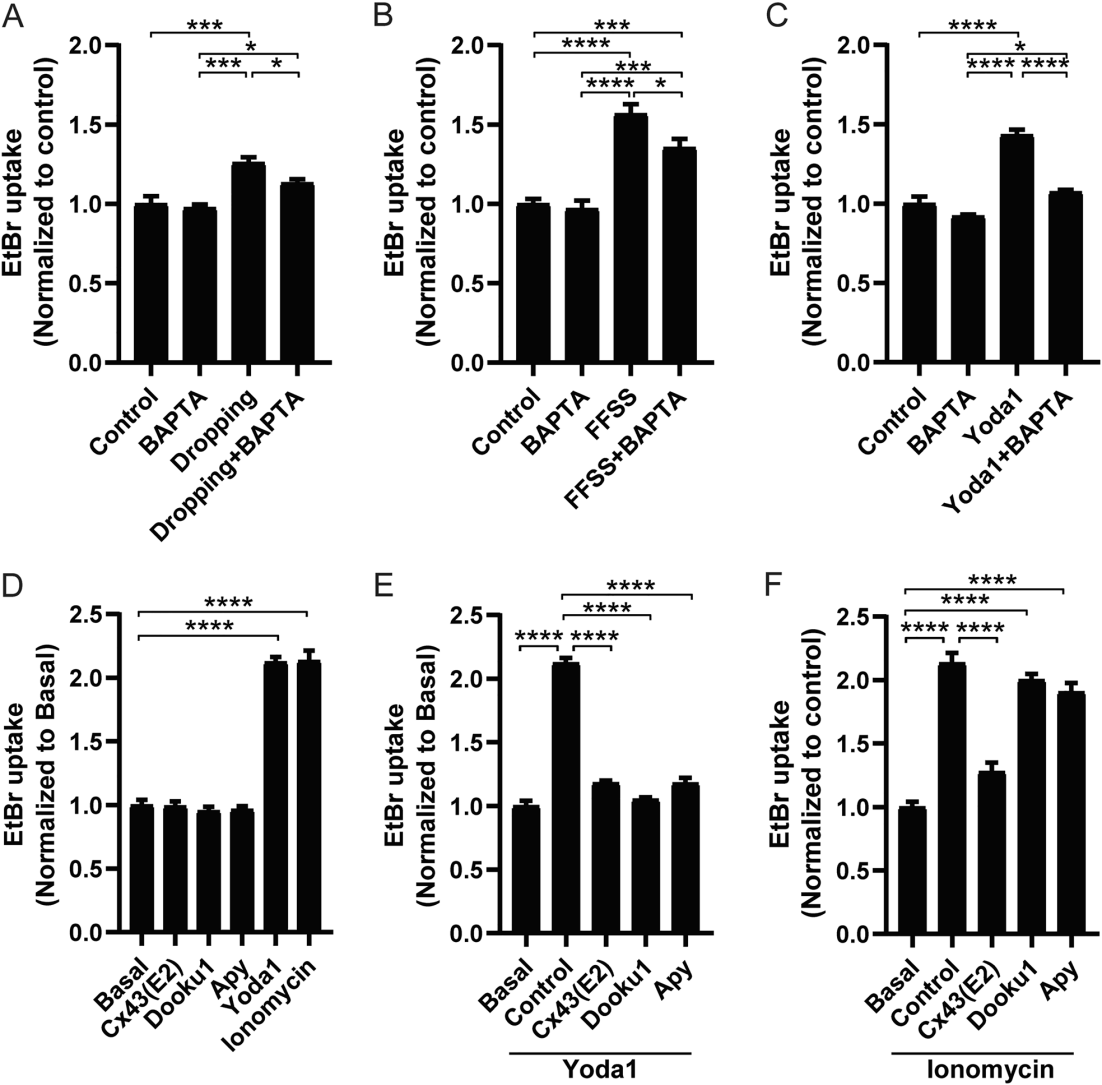
Mechanical loading activated Piezo1 and Ca^2+^ influx induces the opening of Cx43 HCs. **A**, **B**, and **C** EtBr dye uptake of MLO-Y4 cells pre-incubated with BAPTA, followed by fluid dropping (**A**), FFSS (**B**), and Yoda1 (**C**) treatment. **D**, **E**, and **F**) EtBr dye uptake level in MLO-Y4 cells pre-incubated with Cx43(E2) antibody, Dooku1, apyrase or BAPTA, at basal level (**D**) or followed by treatment with Yoda1 (**E**) or ionomycin (**F**); Data are shown as mean ± SEM, n = 3. *p < 0.05, ***p < 0.001, ****p < 0.0001. Statistical analysis was performed using One-way ANOVA for multiple comparison analysis

Intracellular Ca^2+^ dynamics play a crucial role in osteocyte mechanosensitivity [35]. To assess if intracellular Ca^2+^ signal activation by mechanical loading is responsible for the increase of plasma membrane permeability, we pretreated the cells with BAPTA, an intracellular Ca^2+^ chelator, and applied two forms of mechanical loading, fluid dropping and FFSS, and performed dye uptake. The results showed that BAPTA partially inhibited dye uptake induced by fluid dropping (Fig. 3A) and FFSS (Fig. 3B), suggesting that increased membrane permeability is partially dependent on intracellular Ca^2+^. Further, BAPTA inhibited the increase of dye uptake induced by Yoda1, suggesting Piezo1’s role as a Ca^2+^ channel during the rise in plasma membrane permeability (Fig. 3C). In order to understand the mechanism of activated-Piezo1 on increased plasma membrane permeability, we pretreated cells with Cx43(E2) antibody, Dooku1, or apyrase (Apy), an ATP hydrolyzing enzyme, and then applied Yoda1 or ionomycin, a Ca^2+^ ionophore. Both Yoda1 and ionomycin induced dye uptake (Fig. 3D), suggesting that elevated intracellular Ca^2+^ concentration increased the plasma membrane permeability. Dye uptake induced by Yoda1 was significantly inhibited by Dookul and apyrase (Fig. 3E). A comparable level of inhibition on HCs was also shown when we pretreated the cells with Cx43(E2) antibody (Fig. 3E). These results suggest that increased intracellular Ca^2+^ induced by Piezo1 and release of ATP to the extracellular side are required for the activation of Cx43 HCs. However, unlike the partial inhibition mediated by Cx43(E2) antibody, pre-treatment of Dooku1 and Apy did not inhibit HCs uptake induced by ionomycin (Fig. 3F), indicating that increased intracellular Ca^2+^ alone could promote the activity of Cx43 HCs and this activation could be independent of Piezo1 and purinergic receptor pathways. The results suggested that increased intracellular Ca^2+^ by mechanically activated Piezo1 provoked the opening of Cx43 HCs. Furthermore, Piezo1 activation induced the release of intracellular ATP from the osteocytes to the extracellular domain, which is involved in the activation of Cx43 HCs and increase of plasma membrane permeability.

### Intracellular Ca^2+^ signaling activated by Piezo1 regulates the activity of Cx43 HCs and Panx1 channels.

**Fig. 4:**
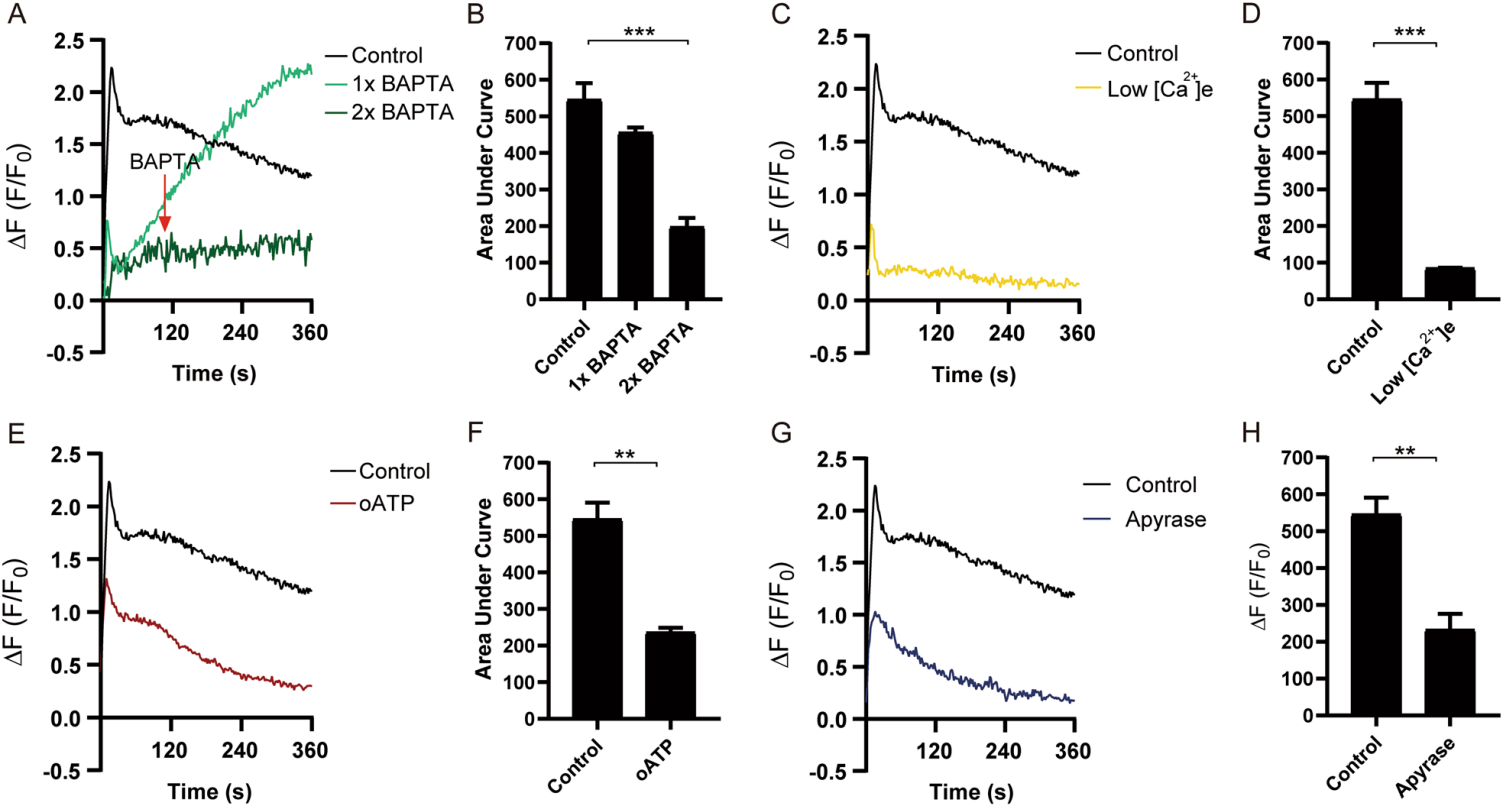
Extracellular Ca^2+^ and P2X receptor signaling are involved in Piezo1 induced elevated intracellular Ca^2+^ signals. **A** and **B** Ca^2+^ signals and AUC of MLO-Y4 cells pre-incubated with BAPTA with/without additional application of BAPTA at 120 s after application of Yoda1. n = 8, 5, and 3 for control, 1xBAPTA, and 2xBAPTA group, respectively. **C** and **D** Ca^2+^ signals and AUC of MLO-Y4 cells under a low extracellular Ca^2+^ environment, followed with Yoda1 treatment, n = 8 and 3 for control and low extracellular Ca^2+^ environment group, respectively. **E** and **F** Ca^2+^ signals and AUC of MLO-Y4 cells pre-incubated with oATP, followed by Yoda1 treatment, n = 5 and 3 for control and oATP group, respectively. **G** and **H** Ca^2+^ signals and AUC of MLO-Y4 cells pre-incubated with apyrase, followed with Yoda1 treatment. n = 5 and 3 for control and apyrase group, respectively; Data are shown as mean ± SEM, *p < 0.05, **p < 0.01. Student-Newman Keul’s test for panels **D**, **F**, and **H**, and One-way ANOVA was used in panel B

Activated Piezo1 promoted the increase of intracellular Ca^2+^, which was necessary to induce dye uptake through the activation of Cx43 HCs and possibly other pathways. To understand the nature of intracellular Ca^2+^ signals activated by Piezo1, MLO-Y4 cells were loaded with Fluo4-AM, then rinsed with recording media, and live cell imaging of free Ca^2+^ was recorded under the microscope. The osteocytes showed a significant increase of free intracellular Ca^2+^ level induced by Yoda1, with a fast peak in the first seconds and a persistent decrease that remains for minutes (Fig. 4A). In cells loaded with 5 μM BAPTA-AM (1 × BAPTA), the transitory increase of free Ca^2+^ levels induced by Yoda1 was totally inhibited; however, the decreased free Ca^2+^ levels recovered gradually and reached an even higher level than control (Fig. 4A). We speculated that the Ca^2+^ pattern was likely caused by saturation or turnover of BAPTA. To mitigate those possibilities, we applied another dose of BAPTA-AM (5 μM, 2 × BAPTA) at the 120 s time point in the recording media. As expected, the second dose of BAPTA-AM suppressed the Ca^2+^ signal to a relatively low and steady level (Fig. 4A). The quantification of the area under the curve (AUC) [36], representing the total level of Ca^2+^ signal, showed the second addition of BAPTA significantly reduced the free Ca^2+^ level induced by Yoda1 (Fig. 4B). Additionally, low extracellular Ca^2+^ condition significantly reduced intracellular Ca^2+^ signal at both the acute phase (first peak) and latent phase (up to 360 s) (Fig. 4C and D), confirming that the extracellular source of Ca^2+^ was necessary to trigger later events. Oxidized ATP (oATP), a potent inhibitor of ATP-activated P2X purinergic receptors (Fig. 4E and F) [37, 38], and apyrase (Fig. 4G and H) showed similar reduced Ca^2+^ signals in both acute and latent phases. These results suggested that ATP release promoted by Piezo1 activation activates purinergic receptors, likely P2X, on the cell surface and induces extracellular Ca^2+^ entry, leading to increased intracellular Ca^2+^ levels.

**Fig. 5:**
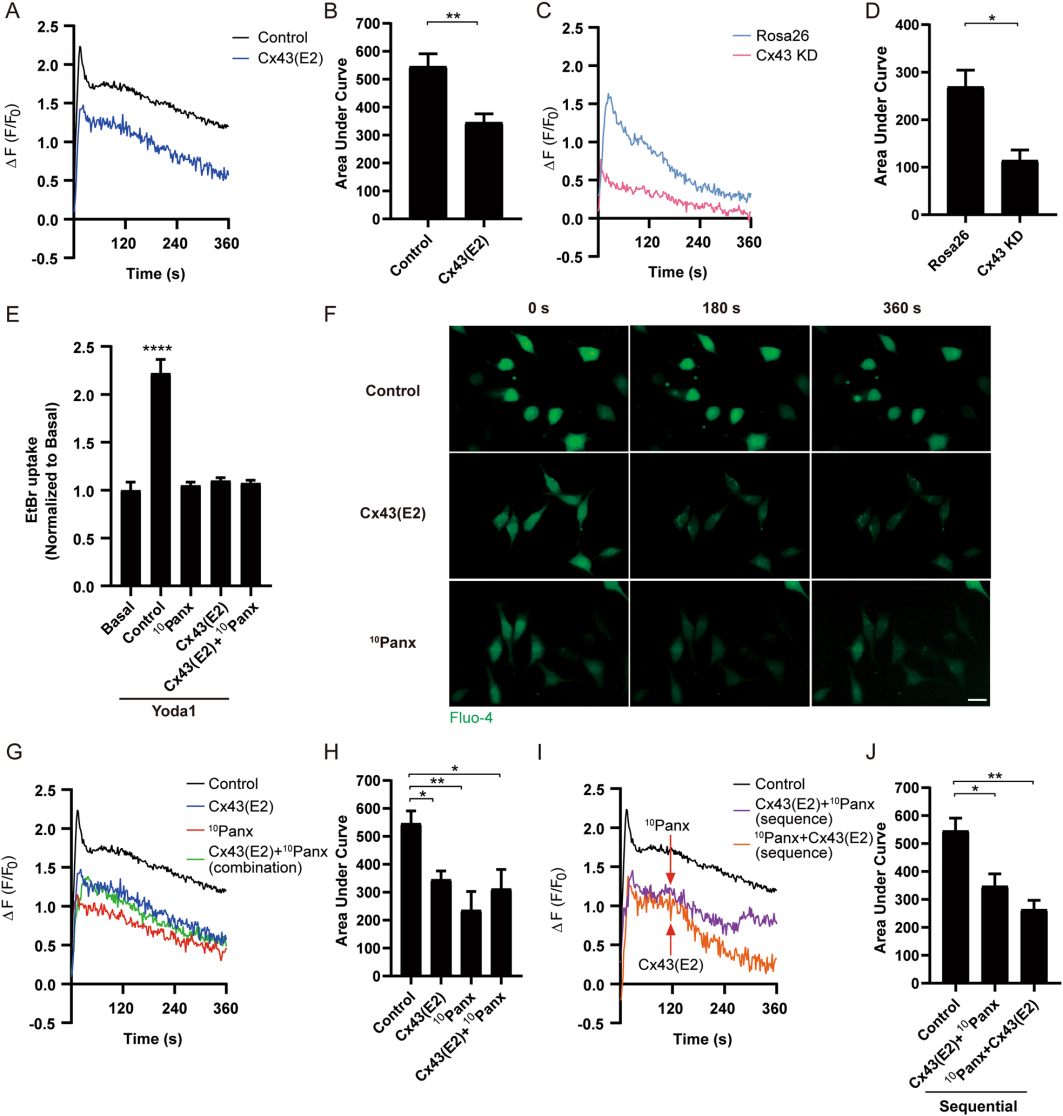
Cx43 HCs and pannexin1 channels regulate intracellular Ca^2+^ signaling activated by Piezo1. **A** and **B** Ca^2+^ signals and AUC of MLO-Y4 cells pre-incubated with Cx43(E2), followed with Yoda1 treatment. n = 8 and 6 for control and Cx43(E2) group, respectively. **C** and **D** Ca^2+^ signals and AUC of Rosa26 and Cx43 KD MLO-Y4 cell, n = 3. **E** EtBr dye uptake on MLO-Y4 cells pre-incubated with Cx43(E2), ^10^Panx, and Cx43(E2) plus ^10^Panx with Yoda1 treatment. n = 6; **F** Representative Ca^2+^ images of Cx43(E2), ^10^Panx, or Cx43(E2) plus ^10^Panx pre-incubated groups at 0 (application Yoda1), 180, and 360 s, **(F)** are representative images from three independent experiments. **G** and **H** Ca^2+^ signals and AUC of MLO-Y4 cells pre-incubated with Cx43(E2), ^10^Panx, and Cx43(E2) plus ^10^Panx, followed with Yoda1 treatment, n = 8, 6, 3, and 3 for control, Cx43(E2), ^10^Panx, and Cx43(E2) plus ^10^Panx group, respectively; **I** and **J** Ca^2+^ signals of MLO-Y4 cells pre-incubated with Cx43(E2) antibody or ^10^Panx with additional 10Panx or Cx43(E2) antibody treatment 120 s after Yoda1 application, respectively, during live imaging, n = 8, 3, 3 for control, and two sequential groups, respectively; Data are shown as mean ± SEM, *p < 0.05, **p < 0.01, ****p < 0.0001. Student-Newman Keul’s test for panels **B** and **D**, and One-way ANOVA for panels **E**, **H**, and **J**

Since Cx43 HCs can mediate extracellular Ca^2+^ influx and ATP release [39–41], we determined if Cx43 HCs are involved in the regulation of intracellular Ca^2+^ evoked by Piezo1 using both Cx43(E2) antibody and Cx43 knockdown. MLO-Y4 cells were pre-incubated with Cx43(E2) antibody (2 μg/ml), then live Ca^2+^ images were captured, and Yoda1 was applied during the imaging. Compared with the control, the intracellular Ca^2+^ signal (first peak) decreased significantly, while exhibiting a parallel declining curve for the rest of the recording time periods (Fig. 5A). By quantifying total levels of Ca^2+^ signals, AUC showed a similar reduction in the Cx43(E2) antibody-treated group (Fig. 5B). Similarly, intracellular Ca^2+^ signals in Cx43 KD MLO-Y4 cells also showed a reduction in the first peak and following time periods (Fig. 5C) and AUC (Fig. 5D). Interestingly, the knockdown of Cx43 was associated with a decreased expression of Piezo1 protein (Additional file 1: Fig. S2), which could partially explain the reduced Ca^2+^ signal peak induced by Piezo1 channel activation compared with MLO-Y4 treated with Cx43(E2) antibody.

We showed that ATP release is involved in the increase of dye uptake. In addition to Cx43 HCs, Panx1 channels are present in osteocytes and can be activated by extracellular ATP [42–44]. We showed that pre-treatment with ^10^Panx (150 μM), an inhibitory mimetic peptide for Panx1 channels, also significantly reduced Yoda1-induced dye uptake (Fig. 5E). To determine the participation of Panx1 channels in intracellular Ca^2+^ dynamics induced by Piezo1 activation, MLO-Y4 cells were pre-incubated with 150 μM ^10^Panx. The representative live images at 0 s (application of Yoda1), 180 s, and 360 s showed that both Cx43(E2) antibody and ^10^Panx could significantly reduce Ca^2+^ signals at both acute phase and latent phase (Fig. 5F). Compared with the control, the Ca^2+^ signaling pattern of ^10^Panx (Fig. 5G, red line) showed a comparable level of the reduction compared to that of the Cx43(E2) antibody (Fig. 5G, blue line) or combination with both Cx43(E2) antibody and ^10^Panx (Fig. 5G, green line). AUC quantification also showed a similar reduction in these treated groups (Fig. 5H). To determine whether Cx43 HCs and Panx1 channels engage at different phases of Ca^2+^ signaling activated by Piezo1, including the acute phase indicated by the first peak and latent phase up to 360 s period, we applied Cx43(E2) antibody and ^10^Panx in sequential order by first preincubating cells with Cx43(E2) antibody (Fig. 5I, purple line) or ^10^Panx (Fig. 5I, orange line) 30 min prior to adding Yoda1 and followed by ^10^Panx (Fig. 5I, purple line) or Cx43(E2) antibody (Fig. 5I, orange line) 120 s after Yoda1 application. Although there was a similar level of reduction at the acute phase by either treatment, the group pre-incubated with ^10^Panx and followed by Cx43(E2) antibody showed a lower level of Ca^2+^ signal (Fig. 5I) and AUC (Fig. 5J) compared to the other treated group. The results suggest that both Cx43 HCs and Panx1 channels are downstream pathways of Piezo1 activation and are engaged in the regulation of intracellular Ca^2+^ influx induced by Piezo1 activation, and Cx43 HCs appear to be more involved in maintaining the latent phase Ca^2+^ signal while Panx1 channels are inactivated earlier than Cx43 HCs.

### Piezo1 co-localizes with Cx43 HCs on osteocyte plasma membrane and activates Cx43 HCs through PI3K-Akt signaling pathway

**Fig. 6:**
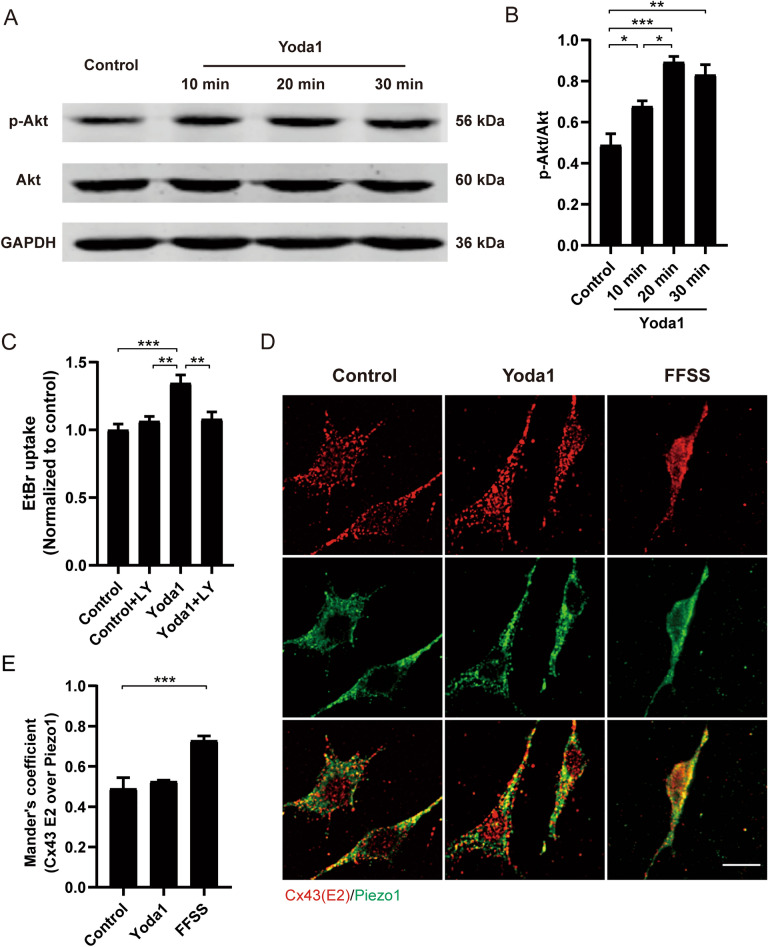
Piezo1 co-localizes with Cx43 HCs on osteocyte cell surface and activates Cx43 HCs through PI3K-Akt pathway. **A** Western blot analysis of p-Akt and Akt protein level form whole lysates of MLO-Y4 cells treated with 20 μM Yoda1 for 10, 20, and 30 min. **B** Quantification of p-Akt/Akt in **A**, n = 3; **C** EtBr dye uptake level of MLO-Y4 cells pre-incubated with 20 μM LY294002, followed treatment with 20 μM Yoda1. n = 3. **D** Representative images out of three independent experiments of cell surface staining on MLO-Y4 cells under control or treated with 20 μM Yoda1 or FFSS (16 dynes/cm^2^) stimulation. (Red for Cx43 and green signal for Piezo1). **E** Mander’s coefficients of Cx43(E2) over Piezo1. Data are shown as mean ± SEM, *p < 0.05, **p < 0.01, ***p < 0.001. Statistical analysis was performed using One-way ANOVA for multiple comparison analysis

We have previously reported that PI3K-Akt signaling pathway regulates the opening of Cx43 HCs in osteocytes [16]. Additionally, mechanical stimulation in osteocytes suppresses Sost expression through the Piezo1-Akt pathway [45]. To assess whether the PI3K-Akt pathway was involved in the Piezo1-induced Cx43 HCs activation, MLO-Y4 cells were incubated with 20 μM Yoda1 for 10, 20, and 30 min and whole-cell lysates were collected for western blot analysis with antibodies against total Akt and phospho (p)-Akt form. The expression of p-Akt started to increase as early as 10 min and reached the highest level around 20 min (Fig. 6A, Additional file 1: Fig. S3A). The normalized levels of p-Akt/Akt showed a similar increase at 10 min, reaching the highest level at 20 min after Yoda1 treatment (Fig. 6B), while Akt expression showed a reduction trend (Additional file 1: Fig. S3B). Preincubation with LY294002 (LY), a PI3K pathway inhibitor, significantly reduced Yoda1-induced EtBr uptake (Fig. 6C, Additional file 1: Fig. S1), showing that Piezo1 activation promoted the PI3K pathway activation and therefore induced the activation of Cx43 HCs in agreement with our previous work [17]. Our previous work revealed the requirement of PI3K signaling from the dendrites, where αvβ3 integrin is located, to the cell body, where functional Cx43 HCs are expressed [16]. The high abundance and homogeneous distribution of Piezo1 in the osteocytes [24, 45] may promote the activation of functional Cx43 HCs in the cell body [16, 46]. We performed confocal immuno-fluorescence to study the subcellular distribution of Piezo1 and Cx43 HCs. Cx43(E2) antibody targeting the extracellular domain of Cx43 [29] was used to label the plasma membrane Cx43 in non-permeabilized live cells. The reactivity observed for Cx43 was uniformly distributed over the osteocyte surface (Fig. 6D in red). The localization of Piezo1 was determined in permeabilized cells, because the Piezo1 antibody epitope is intracellular and staining pattern appeared to be homogeneous and reticular (Fig. 6D in green). After treatment with Yoda1 or FFSS (16 dynes/cm^2^, 1 h), there was an increased colocalization of Piezo1 and Cx43 (Fig. 6D, yellow) after FFSS, but increased colocalization signal was not observed with Yoda1 treatment. The level of localization was significantly higher after FFSS using Mander’s analysis, with averaged coefficients of 0.73 for FFSS and 0.50 for the remaining conditions (Fig. 6E). Interaction between Piezo1 and Cx43 was also demonstrated by immunoprecipitation of Cx43 by Piezo1 antibody (Additional file 1: Fig. S4). Together, these results suggested that FFSS enhanced the co-localization of Piezo1 and Cx43 HCs at the osteocyte plasma membrane. Furthermore, the PI3K-Akt pathway is involved in the Piezo1-induced opening of Cx43 HCs.

### Piezo1 activated by mechanical loading induces the opening of Cx43 HCs in bone in vivo

**Fig. 7:**
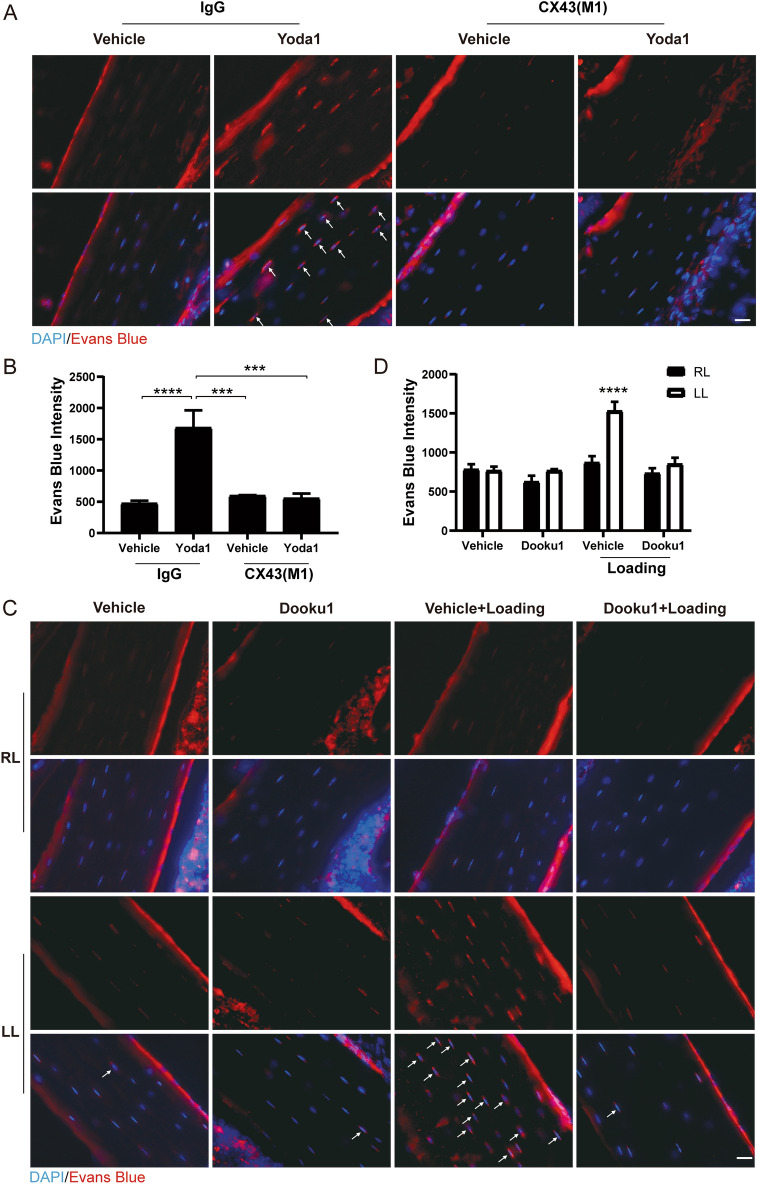
Piezo1 is activated by mechanical loading and induces the opening of Cx43 HCs in vivo. **A** and **B** 15-week-old male C57BL/6 J mice were treated with M1, IgG, or/and Yoda1, or vehicle DMSO and Evan blue dye. Representative images of tibia frozen sections of Evans blue uptake (**A**) and quantification of Evans blue intensity (**B**), n = 5 per group; **C** and **D** 15-week-old male C57BL/6 J mice were treated with Cx43(M1), IgG, or/and Dooku1, or DMSO and tibia compression and Evan blue dye. Representative images of Evans blue uptake (**C**) and quantification of Evans blue intensity (**D**). n = 5 per group. Data are shown as mean ± SEM, *** *p* < 0.001, ****p < 0.0001; White arrows represent osteocytes uptaking Evans Blue. Statistical analysis was performed using One-way ANOVA for multiple comparison analysis for panel **B** and Two-way ANOVA between multiple groups with two variables for panel **D**

To demonstrate if the mechanism concerning the activated Piezo1 by mechanical stimulation in Cx43 HCs opening could be recapitulated in osteocytes in situ, we injected into 15-week-old male C57BL/6J mice an HC-blocking Cx43(M1) antibody, a mouse monoclonal antibody that binds to the extracellular domine of Cx43 and inhibits HCs [15], or the same amount of mouse IgG and performed Evans blue uptake assay with the administration of Yoda1. Bone tissue sections were taken around the tibial midshaft region and the results suggested that Yoda1 induced Cx43 HCs opening in tibial bone in vivo, and this opening was inhibited by Cx43(M1) antibody (Fig. 7A). Quantification of Evans Blue signals showed the increased dye uptake by Yoda1 was significantly inhibited by Cx43(M1) antibody (Fig. 7B). To evaluate the participation of Piezo1 channels during mechanical loading in vivo, mice were subjected to tibial compression on the left tibia, and the right tibia served as a contralateral non-loaded control. Evans blue uptake of osteocytes around the midshaft region (55% tibia length proximal to distal) was increased by tibial compression and this increase was significantly inhibited with the administration of Dooku1 (Fig. 7C and D). The results demonstrated that Cx43 HCs opening in bone osteocytes is mediated through mechanical loading activated Piezo1 both in vitro and in vivo.

## Discussion

**Fig. 8:**
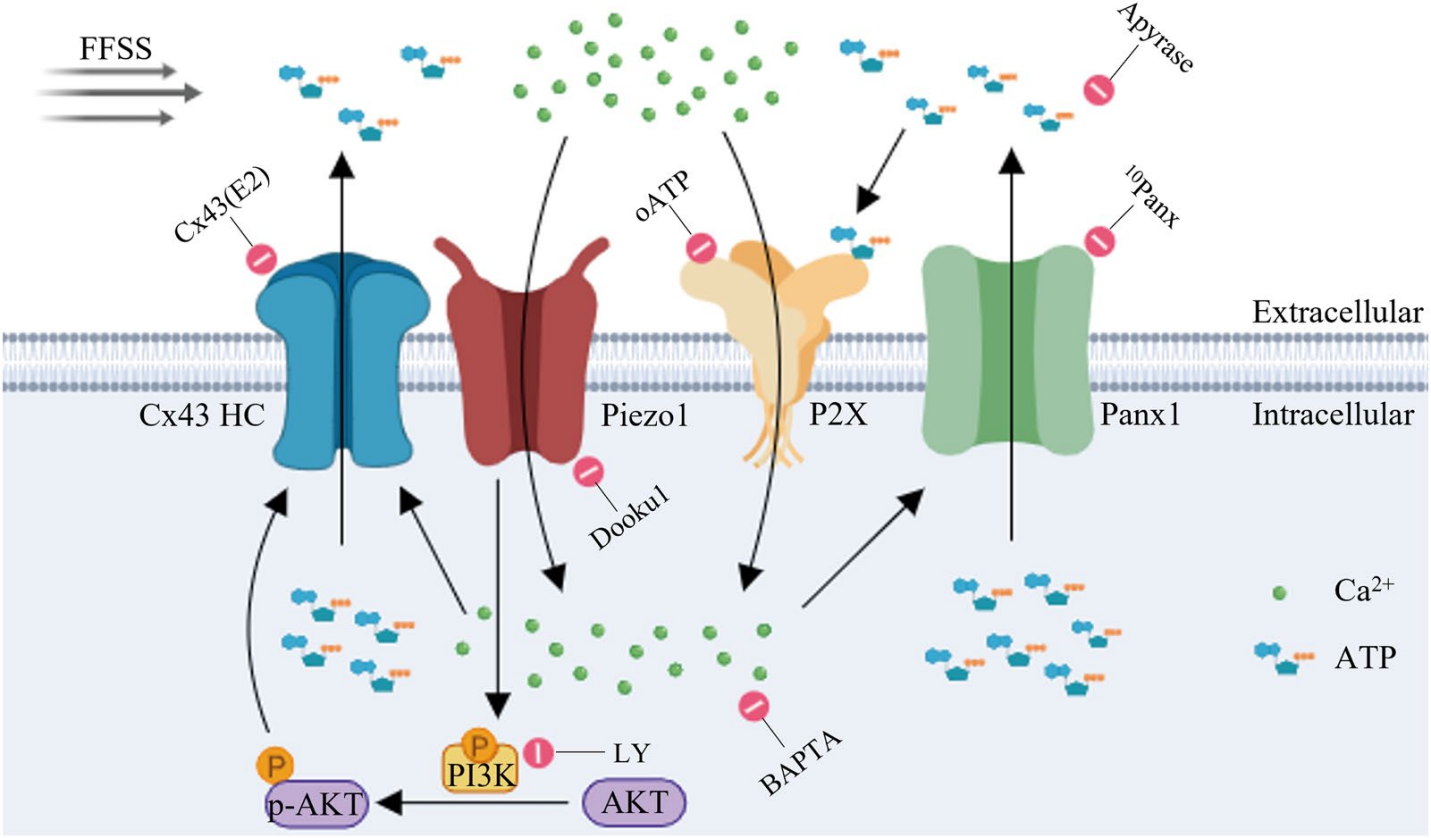
Illustration of mechanical loading activated-Piezo1 induces opening of Cx43 HCs via PI3K-Akt pathway. Fluid flow shear stress (FFSS) induces the activation of the Piezo1 Channel, increasing the intracellular Ca^2+^ levels, leading to an increase in the activity of both Cx43 HCs and Panx1 channels, allowing the release of ATP. Extracellular ATP activates P2X receptors promoting the entrance of Ca^2+^ into the cytoplasm, holding the activity of Cx43 HCs and Panx1 channels. Active Piezo1 Channel complex could lead to the activation of the AKT pathway and stabilization of active Cx43 HCs in the cell surface. This system could be inactivated by the action of extracellular phosphatases and reducing the ATP levels. The inhibition of Piezo1 channels (Dooku1) and/or AKT pathway (LY) close Cx43 HCs

Osteocytes are the pivotal mechanosensitive and mechano-transducing bone cells in sensing the mechanical stimulations and regulating bone formation and remodeling [2]. Gap junctions, which play an essential role in intracellular communication, present a classic formation paradigm between two adjacent cells, and allow the direct transfer of critical small metabolites, ions, and signaling molecules. Different from gap junctions, unpaired Cx43 HCs allow the exchange of small molecules with extracellular milieu and orchestrate bone formation and remodeling processes [7, 47]. We found in our previous studies that Cx43 HCs are an essential portal for the release of PGE_2_, a critical anabolic factor in response to mechanical stress [8]. More recently, we showed that impaired Cx43 HCs compromised bone formation in response to tibial loading [15]. The piezo1 subtype is mainly expressed in non-excitable cells, including skeletal tissues [48]. The unique feature of the Piezo1 channel is that it serves as the mechanosensitive and mechanotransductive channels in bone, the organ which is highly sensitive to mechanical stimulation and the metabolic regulation of which is a lifelong event starting from the embryonic stage [49]. Genetic mouse studies regarding Piezo1 function in bone homeostasis demonstrate that Piezo1 mediates cellular mechanosensation in mesenchymal stem cells, osteoblasts, and osteocytes [25, 49]. In this study, we, for the first time, demonstrated a close relationship between these two types of channels in osteocytes. We showed that Piezo1 specific agonist Yoda1 could increase the plasma membrane permeability, inducing the opening of Cx43 HCs in a dose and time-dependent manner. In addition, we also unveiled a new signaling pathway where active Piezo1 channels trigger the increase of intracellular Ca^2+^ levels, leading to the opening of Cx43 HCs and Panx1 channels. Moreover, Piezo1 activation leads to the activation of the PI3K-Akt signaling pathway required for the opening of Cx43 HCs. We also observed the colocalization of Piezo1 and Cx43 HCs, suggesting the proximity of the two proteins, and this co-localization was enhanced by mechanical stimulation. Furthermore, we found that the activity of Cx43 HCs along with Panx1 channels and the activation of P2X receptors was necessary to further elevate intracellular Ca^2+^ levels initiated by Piezo1 channels. The role of Piezo1 in regulating Cx43 HC opening by mechanical loading was also confirmed in osteocytes of tibial bone in situ. Together, as illustrated in the diagram (Fig. 8), Piezo1 channels activated by mechanical loading increased intracellular Ca^2+^, which promotes the activation of Cx43 HCs and Panx1 channels. These active channels facilitate the release of ATP, leading to the activation of P2X receptors. The ATP receptor activation could generate more Ca^2+^ influx, thus recruiting more Cx43 HCs and Panx1 channels to sustain the intracellular Ca^2+^ signaling initiated by Piezo1 channel activation. The elevated activity of the PI3K-Akt pathway, activated by Piezo1, increased intracellular Ca^2+^ and further enhanced the opening of Cx43 HCs.

We confirmed the involvement of Panx1 channels using ^10^Panx, a specific mimetic peptide that blocks Panx1 channel activity [50]. In addition to Cxs, Panxs are high capacity channels, permeable to ions and small molecules, and share similar pharmacological properties and membrane topology with Cx HCs [33]. However, they are not homologous to Cxs in their gene and protein sequences and do not share structural features with Cx channels [51]. Panx1 channels like Cx43 HCs mediate the release of ATP and therefore regulate Ca^2+^ wave propagation and apoptosis through its interplay with the P2X7 purinergic receptor [52]. The interplay of Panx1-released ATP and P2X7 receptors leads to large pore formation and induces dye uptake [53]. We showed that inhibition of the Panx1 channel by ^10^Panx decreased dye uptake to a comparable extent to Cx43(E2) antibody; however, a greater level of reduction was not observed by combined treatment, probably due to the presence of other pathways that uptake the dye and are sensitive to ^10^Panx. Interestingly, the action of Cx43 HCs and Panx1 channels was not additive, but interconnected, requiring each other to be active. Several studies have shown that P2X receptor activation is unaffected by genetic deletion or pharmacological inhibition of Panx1 [54], which indicates that the function of purinergic P2X receptor may be independent of Panx1, and the inhibitory effect of ^10^Panx on Panx1 channels may not affect P2X receptors [50].

The acute event that takes place within 1 min of mechanical stimulation is the increase of intracellular Ca^2+^, which is required for the following ATP release [4]. Our data suggest that the initial step involves the activation of Piezo1 channels by mechanical loading accompanied by the increase of the intracellular Ca^2+^ concentration. Previous studies proved that the opening of Cx43 HCs is triggered by moderate intracellular Ca^2+^ concentration rises (≤ 500 nM), and HCs opening allows the influx of Ca^2+^ [55]. Based on Ca^2+^ imaging results, opening of Cx43 HCs is believed to occur at a later phase, while Piezo1 channel activation is responsible for the initial intracellular Ca^2+^ peak. Moreover, we also observed that the opening of Cx43 HCs allows the release of molecules like ATP that could activate P2X channels expressed on the cell surface, suggesting an indirect Ca^2+^ signaling pathway for the sustained influx of Ca^2+^ mediated by Cx43 HCs (Fig. 4E, G). Increased intracellular Ca^2+^ could recruit and trigger the activation of Cx43 HCs and Panx1 channels, leading to ATP release [41, 56]. ATP activated purinergic signaling leads to the activation of downstream events, including activation of the P2X receptor and Panx1 channels. Another important event is the FFSS-activated PI3K pathway, which also induces Cx43 HCs opening and the stabilization of Cx43 HCs on the cell surface [6, 8, 17, 44, 57]. Previous studies have shown that osteocytes exhibit repetitive Ca^2+^ spikes, more commonly known as Ca^2+^ oscillation, in response to dynamic loading [58]. Endoplasmic reticulum (ER) Ca^2+^ channel and T-Type voltage sensitive Ca^2+^ channels (VSCC) are also involved in the regulation of cytoplasmic Ca^2+^ (Ca^2+^_cyt_). The Ca^2+^_cyt_ and Ca^2+^_ER_ oscillation was subtly synchronized, and the increased Ca^2+^_cyt_ was proven to refill Ca^2+^_ER_, and the T-Type VSCC facilitates this behavior [35]. However, the regulatory role of HCs in Ca^2+^ signaling under mechanical stimulation remained largely elusive. We investigated intracellular Ca^2+^ signals by live imaging upon mechanical loading and activated Piezo1. Unlike the Ca^2+^ oscillation pattern on a single cell, in this study, the averaged Ca^2+^ signal of 18 to 36 cells from 3 to 6 independent experiments showed a relatively consistent pattern. The difference might be due to the delayed synchronization between cells, as well as the calculated Ca^2+^ signal derived from combined Ca^2+^_cyt_ and Ca^2+^_ER_. Interestingly, the inhibition of Cx43 HCs and Panx1 both decrease intracellular Ca^2+^ signals at both acute and latent phases.

To explore the temporal relationship of Cx43 HCs and Panx1 channels, we inhibited these two channels in a sequential manner. Despite the similar reduction of the first peak, the application of Cx43(E2) antibody at the later phase showed a lower Ca^2+^ signal, which may indicate that Cx43 HCs engage more in sustaining later phase intracellular Ca^2+^. Regardless of the sequential order of the inhibitors being applied, a similar, although slightly greater, inhibition of intracellular Ca^2+^ activated by Piezo1, was shown by inhibiting Panx1 channels. It has been known that elevated intracellular Ca^2+^ activates both types of channels [44]. These data indicate that acute increased intracellular Ca^2+^ is likely to be a result of the opening of both Cx43 HCs and Panx1 channels that subsequently mediate Ca^2+^ increase. The slightly greater reduction by first applying the Panx1 inhibitor may indicate that the Panx1 channel contributes slightly more to the overall intracellular Ca^2+^ level. A limitation of the study is the time resolution since 2 s time intervals could be relatively too long to resolve the temporal difference attributed by Cx43 HCs and Panx1 channels upon Piezo1 activation. The dye uptake data suggest a functional dependency between Cx43 HCs and Panx1 channels. Similar to the inhibitory effect by Cx43 HCs and Panx1 channels, oATP and apyrase reduced intracellular Ca^2+^ at both acute and latent phases, which indicates the involvement of ATP-activated P2X receptor signaling in regulating intracellular Ca^2+^ dynamics triggered by Piezo1. Moreover, we also observed that apyrase also inhibited HCs dye uptake. These results suggest that a high local concentration of extracellular ATP released, possibly by osteocytic HCs formed by Cx43 and Panx1 channels, activates purinergic receptors, leading to further opening of HCs and Ca^2+^ influx at the latent phase.

We showed that Yoda1 increased Akt activation, and inhibition of PI3K-Akt signaling inhibited HCs induced by Yoda1. Previous studies reported a close relationship between Ca^2+^ regulation and the PI3K-Akt signaling pathway. Ca^2+^ influx induces the Ca^2+^ release from the endoplasmic reticulum via IP3R activation [59]. The cytosolic release of Ca^2+^ activates PI3K-Akt, which modulates GSK3β activity and therefore regulates the Wnt/β-catenin pathway [60]. Moreover, we have previously shown that activation of the PI3K-Akt pathway plays a critical role in Cx43 phosphorylation and the opening of Cx43 HCs in response to mechanical loading [17]. We recently reported that integrin αVβ3 at dendritic processes senses shear stress, transmits the signal to the cell body by activating intracellular PI3K-Akt signaling, and activates α5β1 and, therefore, opens Cx43 HCs [16]. Here, we demonstrated that the PI3K-Akt pathway is involved in the Piezo1-induced Cx43 HCs opening.

Before the discovery of Piezo1 as a mechanosensitive ion channel, Piezo1 was reported to be involved in integrin activation by recruiting the small GTPase R-Ras to the ER [61]. The Piezo related integrin activation mainly takes part in the regulation of cell migration, such as in endothelial cells and cancer cells [62]. A recent study revealed that the force-dependent co-localization of Piezo1 to β3 integrin could be synergistic with downstream events and provides a mechanism of tension-dependent vascular growth that is critical in the development process and cancer [63]. Other evidence also suggested a close relationship between Piezo1 and β1 integrin [61, 64]. We showed earlier that Cx43 associates with α5β1 integrin [65]. It is plausible that Cx43 and Piezo1 may be co-localized and interact in a complex. Indeed, our immunofluorescence study showed that Piezo1 and Cx43 HCs were co-localized on the cell surface, and this colocation was enhanced upon FFSS. The knockdown of Cx43 also led to a reduction of Piezo1 protein expression, and the interaction between Cx43 and Piezo1 was further confirmed with an immunoprecipitation study. It has been reported that FFSS could increase Piezo1 and Cx43 HCs expression [25]. However, we found the increased co-localization occurred only after 1 h of FFSS, a time frame not sufficient for increased protein synthesis. This observation was likely due to the fact that FFSS increased trafficking and interaction of Piezo1 and Cx43 HCs on the plasma membrane. Interestingly, such enhancement of cell surface co-localization was not observed with the activation of Piezo1 by Yoda1. This possible difference could be due to how the cells were activated; FFSS increases membrane localization of Piezo1 and Cx43, while Yoda1 specifically activates Piezo1 through increased intracellular Ca^2+^ and open HCs. In summary, it is likely that the interplay of Piezo1 and integrin activation upon mechanical loading activates the PI3K-Akt signaling pathway that induces the opening of Cx43 HCs [16, 45, 66],

It is well accepted that fluid flow through the bone lacuna-canalicular system generates shear stress, and therefore in vitro FFSS models have been developed to mimic in vivo shear stress. However, under in vivo situations, the number of dendrites per cell, the changes in lacunar and canalicular number with age, and the configuration of osteocytes vary significantly [67]. Here, we further validated the role of mechanical loading activated Piezo1 in opening Cx43 HCs in vivo. Yoda1-induced Cx43 HCs opening in tibial bone was inhibited by Cx43(M1) antibody and tibial compression induced Evans Blue uptake was significantly inhibited by Dooku1. Previous studies have shown that the Evans blue uptake is a result of increased membrane permeability instead of membrane damage [68]. We have previously used Evans blue to assess HC activity in osteocytes in the bone in vivo [16]. Taken together, Cx43 HCs opening in bone osteocytes is mediated through mechanical loading activated Piezo1 both in vitro and in vivo.

Recent studies also revealed that mechanical stimulation of osteocytes activates Ca^2+^-dependent contractions and enhances the production and release of extracellular vesicles (EVs) containing bone regulatory proteins [69]. Further study is needed to determine the functional relationship between Piezo1-Cx43 HCs calcium signal and other Ca^2+^-related mechanisms such as T-Type VSCC, Ca^2+^_ER,_ and EVs under mechanical loading conditions and to investigate the structural relationship between Piezo1, integrin, and Cx43 HCs, as well as the underlying mechanism, intracellular Ca^2+^ influx, and activated PI3K-Akt pathway. Together, our findings established a critical relationship between major players in the anabolic function of mechanical loading on bone, Piezo1, Cx43, and Panx HCs.

## Supplementary Information

**Additional file 1: Figure S1**. (A) Western blot analysis of Cx43 and Piezo1 protein expression of Rosa26 and Cx43 KD in MLO-Y4 cell lysate. (B and C) Western blot quantification of p-Akt and Akt protein expression of different duration (10, 20, and 30 min) of Yoda1 treatment in lysates of MLO-Y4 cells, n=3 from three independent experiments. Data are shown with mean ± SEM, *p < 0.05, **p < 0.01, ***p < 0.001. Statistical analysis was performed using One-way ANOVA for multiple comparison analysis. (D) Representative EtBr/FITC-dextran dye uptake images of MLO-Y4 cells pre-incubated with LY294002, followed with Yoda1 treatment from three independent experiments, scale bar=20 μm; (E) Cell lysates and immunoprecipitants with Piezo1 antibody were immunoblotted with Cx43 or Piezo1 antibody.

## Data Availability

Not applicable.

## Abbreviations

Cx43 HCs: Connexin 43 hemichannels
FFSS: Fluid flow shear stress
Panx1: Pannexin1
PGE_2_: Prostaglangdin E_2_
NO: Nitric oxide
EtBr: Ethidium bromide
RT: Room temperature
SDS: Sodium dodecyl sulfate
Cx43 KD: Connexin 43 knockdown
CBX: Carbenoxolone
AUC: Area under the curve
oATP: Oxdized ATP
Ca^2+^_cyt_: Cytoplasmic Ca^2+^
Ca^2+^_ER_: Endoplasmic reticulum Ca^2+^
EVs: Extracellular vesicles
