# Supplementary material for: Mechanosensitive piezo1 calcium channel activates connexin 43 hemichannels through PI3K signaling pathway in bone
Source: Cell Biosci. 2022 Dec 1;12:191. doi: 10.1186/s13578-022-00929-w (PMC9716748; doi:10.1186/s13578-022-00929-w)
Supplement: Supplementary file 1 — Additional file 1: Figure S1. (A) Western blot analysis of Cx43 and Piezo1 protein expression of Rosa26 and Cx43 KD in MLO-Y4 cell lysate. (B and C) Western blot quantification of p-Akt and Akt protein expression of different duration (10, 20, and 30 min) of Yoda1 treatment in lysates of MLO-Y4 cells, n=3 from three independent experiments. Data are shown with mean ± SEM, *p < 0.05, **p < 0.01, ***p < 0.001. Statistical analysis was performed using One-way ANOVA for multiple comparison analysis. (D) Representative EtBr/FITC-dextran dye uptake images of MLO-Y4 cells pre-incubated with LY294002, followed with Yoda1 treatment from three independent experiments, scale bar=20 μm; (E) Cell lysates and immunoprecipitants with Piezo1 antibody were immunoblotted with Cx43 or Piezo1 antibody. [file 13578_2022_929_MOESM1_ESM.pdf]

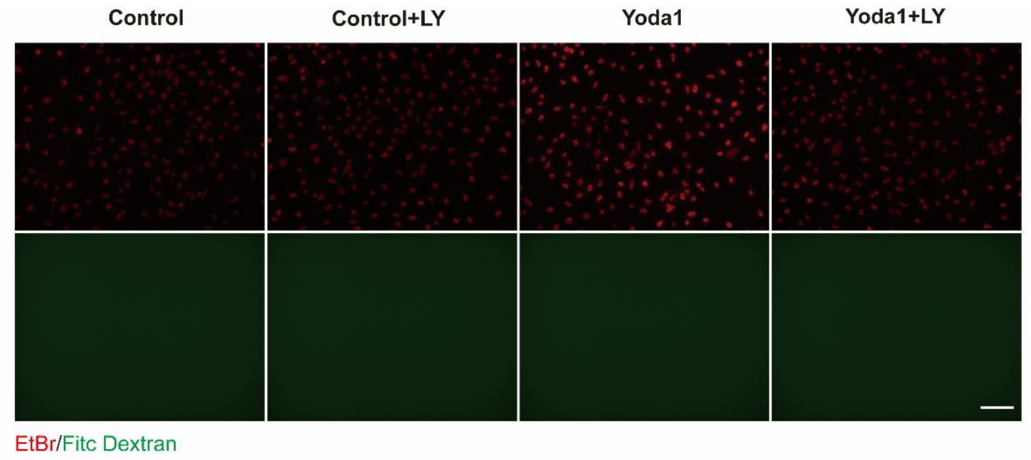

Fig. S1. Piezo1 activates Cx43 HCs through PI3K-Akt pathway. Representative EtBr/Fitc-dextran dye uptake images of MLO-Y4 cells pre-incubated with LY294002, followed with Yoda1 treatment, Fig. S1 are representative ones from three independent experiments, scale bar=20 μm.

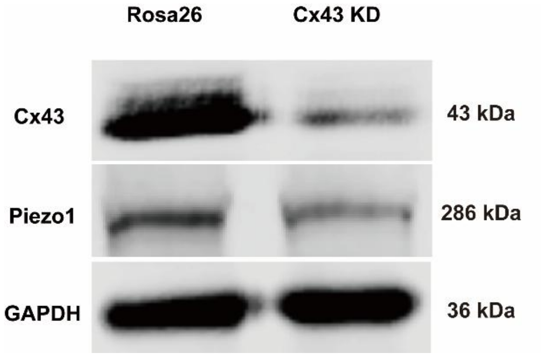

Fig. S2. Piezo1 protein level of Cx43 KD MLO-Y4 cells. Western blot analysis of Cx43, Piezo1, and GAPDH protein expression of Rosa26 and Cx43 KD MLO-Y4 cell lysate. Fig. S2 are representative ones from three independent experiments.

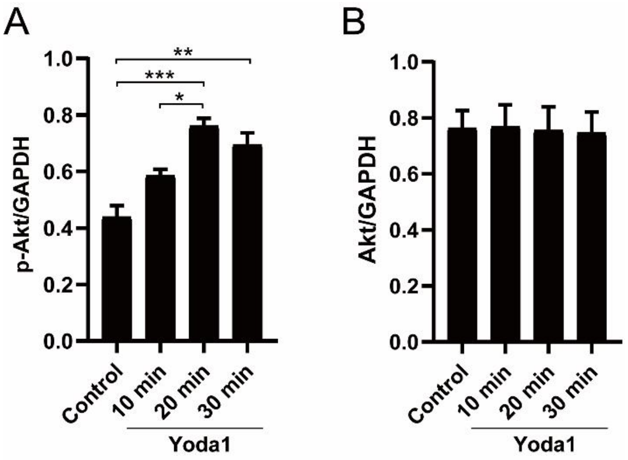

Fig. S3. Piezo1 activates Cx43 HCs through PI3K-Akt pathway. Western blot quantification of p-Akt (A) and Akt protein expression of different duration (10, 20 and 30 min) of Yoda1 treatment in lysates of MLO-Y4 cells, n=3 from three independent experiments. Data are shown with mean ± SEM, *$p < 0.05$, **$p < 0.01$, ***$p < 0.001$. Statistical analysis was performed using One-way ANOVA for multiple comparison analysis.

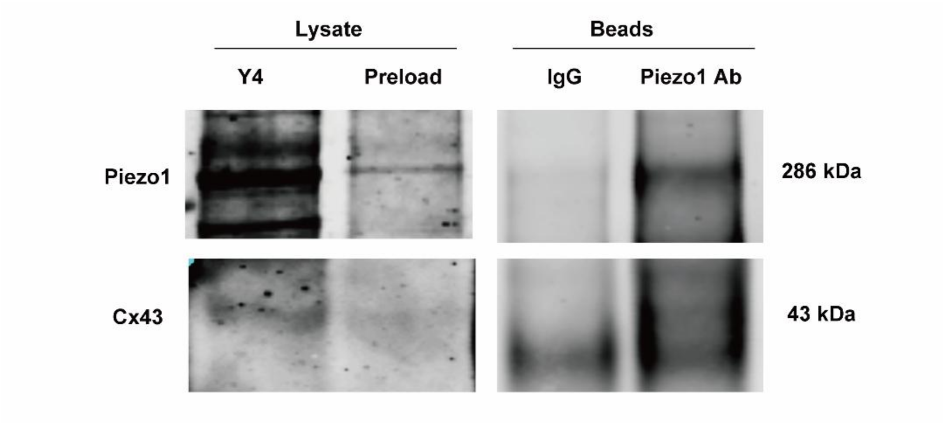

Fig. S4. Piezo1 co-localizes with Cx43 HCs on osteocyte cell surface. Cell lysates and immunoprecipitants with antibody to Piezo1 were probed for Cx43 or Piezo1.
